# Differences in help-seeking behaviour between males and females with multiple pelvic floor symptoms: A qualitative study

**DOI:** 10.1016/j.heliyon.2024.e29110

**Published:** 2024-04-03

**Authors:** Kim Groot Wesseldijk, Hannah E. van Reemst, Ellen ter Horst, Grietje E. Knol-de Vries, Marco H. Blanker

**Affiliations:** Department of Primary and Long-term Care, University Medical Center Groningen, University of Groningen, Groningen, the Netherlands

**Keywords:** Primary health care, General practice, Pelvic ﬂoor, Help-seeking behaviour, Urinary incontinence

## Abstract

**Background:**

Pelvic floor symptoms (PFS) are common and frequently co-occur, but few patients seek help despite negative effects on their quality of life. Moreover, most studies assessing help-seeking behaviour have only focused on a single PFS.

**Aim:**

We aimed to explore the barriers to and facilitators of help-seeking behaviour in males and females with at least two PFS.

**Design and Setting:**

This interview-based study included participants by age and symptoms (number and type) from a larger group taking part in a survey on PFS in the general population.

**Method:**

Two researchers independently encoded and analyzed the semi-structured interviews, which continued to saturation for both the male and female cohorts.

**Results:**

Of the 25 participants (13 male and 12 female), 9 sought help for all PFS, 10 did not seek help for any PFS, and 6 sought help for some PFS. We identified themes in domains related to the patient, healthcare professional, environment, and symptoms. Although most themes applied to both sexes, some had greater sex specificity.

**Conclusion:**

Males and females have more similarities than differences in help-seeking behaviour. Healthcare providers should know that patients who seek help for one symptom probably have multiple PFS that the patient has not reported.

## 1 Introduction

Pelvic floor symptoms (PFS) are common in males and females [1,2]. They include lower urinary tract symptoms (LUTS), urinary incontinence (UI), anorectal dysfunction, sexual dysfunction, lower urinary tract pain, and other pelvic pain, as well as pelvic organ prolapse (POP) in females, and they often co-occur [3,4]. Almost one in four females suffer from at least one PFS [5,6]. By contrast, the few studies that exist in male populations have focused on sexual problems and LUTS, thereby neglecting other PFS [3]. Moreover, although PFS can negatively affect quality of life [7], most patients do not seek help and there is a low utilization rate for pelvic floor care [8,9]. Research indicates that about half of all females with UI do not seek help for their PFS despite wanting treatment [[10], [11], [12]], while only 42% of males seek help for LUTS [13]. Fewer than 25% of males and females with sexual symptoms seek the help of a medical professional [14]. The level of help-seeking behaviour for POP ranges from 21.3% to 73.4%, with the lower levels in low-income countries [15].

Help-seeking behaviour is multifaceted but can be defined as an intentional action to solve a problem that challenges a person's ability to function normally [16]. The process begins with the recognition and defining of a problem, which leads to a decision about whether to act [16]. Different psychosocial factors serve as barriers to, or facilitators of, seeking help for PFS. Barriers include embarrassment, thinking the condition is not serious enough, or perceiving PFS as a normal part of aging [12,17]. Facilitators include experiencing bothersome or worsening symptoms and knowing that treatment options exist [12,17]. However, most studies of help-seeking behaviour for PFS have been performed in female populations [[17], [18], [19]], with only a few assessing possible sex and gender differences [11,12]. Notably, most studies in this field have also been limited by having a focus on only one PFS, predominantly studying UI in females and LUTS in males [[11], [12], [13],^18^,^19^].

This study aimed to explore the differences between males and females with at least two PFS in terms of the barriers and facilitators associated with help-seeking behaviour.

## 2 Method

### 2.1 Study design

This qualitative interview-based study was conducted as part of a prospective, observational, population-based cohort study evaluating PFS in the general population of a municipality in the north-eastern part of the Netherlands [4]. Males and females aged ≥16 years were invited through their general practitioner to complete a questionnaire on PFS as part of the cohort study. For this interview-based study, we purposively included those with at least two PFS if they consented to take part in sub-studies, basing selection on age, number and type of symptoms, and help-seeking status (categorized by whether they sought help for all, none, or some PFS). Participants were invited using personalized letters that included a consent form and information about the proposed interview study. All participants gave written informed consent prior to their interview and received a €20 gift card for participation. Our institutional medical ethics committee approved the study.

### 2.2 Cohort study questionnaire

We used several questionnaires to identify symptoms. LUTS were identified using the International Consultation on Incontinence Modular Questionnaire (ICIQ)-male and female (ICIQ-MLUTS/FLUTS) [20]. Defecation problems were determined by the combined Wexner score (Wexner incontinence score plus Wexner constipation score), based on the Groningen Defecation and Fecal Continence (DeFeC) questionnaire [21]. Sexual dysfunction was determined with the Pelvic Organ Prolapse/Incontinence Sexual Questionnaire, IUGA-Revised (PISQ-IR), and Sexual Health in the Netherlands questionnaire, and the ICIQ-MLUTSsex [20,[22], [23], [24]]. Sexual dysfunction in males was indicated by reports of erectile difficulties, ejaculation problems, or pain during intercourse or ejaculation, and in females, by reports of orgasmic dysfunction or problems, vaginismus, vaginal dryness, or pain during intercourse. Pelvic pain was simply defined as an affirmative response to a question about pain in the pelvic region. Finally, POP was considered present if females answered in the affirmative to four of six items on the Pelvic Organ Prolapse Distress Inventory 6 (POPDI-6) [25]. Details about the questionnaires can be found in our publication of the prospective cohort study [4]. Educational level was divided into three categories: lower, medium and higher education, according to the Dutch Central Bureau of Statistics (CBS).

### 2.3 Procedure

Appointments were scheduled for telephone interviews only after receiving written informed consent. An interview guide was developed prior to the interviews. This interview guide was pilot tested by conducting two pilot interviews (1 male, 1 female participant). Two trained female researchers (EH, HR) who had no prior relationships with the participants then conducted the semi-structured interviews with the participants, lasting around 30 min. These aimed to facilitate the open expression of opinions, the emergence of new information, and the evaluation of cognitions related to help-seeking behaviour. At the start of the interview it was verified whether the symptoms, indicated by the participant on the questionnaire, were still present. During the interviews notes were taken to ensure that all questions were asked and to help provide a summary at the end. All interviews were recorded digitally and transcribed verbatim, and the results were checked by respondent validation. The interview guide was adjusted after three interviews. Interviews continued to saturation, when no new themes arose. Data saturation was discussed with two researchers (MB, GKV) who had not performed the interviews.

### 2.4 Data analysis

Thematic content analysis was conducted. Two researchers separately encoded the transcripts using ATLAS.ti [26]. Coding was done sequentially by two researchers (EH and HR for male interviews, HR and KGW for female interviews). Interviews were coded first by the researcher who interviewed the participants. The second researcher continued with the provided codes. Furthermore, they interviewed males first, and used the codes from these interviews as the basis of the interviews with females. Coding differences were discussed by two other researchers (MB, GKV) until consensus was reached. The data, including themes, subthemes and the associations were discussed twice within the study group.

## 3 Results

We interviewed 13 males and 12 females with mean ages of 68.7 ± 12.1 years and 63.4 ± 9.5 years, respectively. Of these, 9 (4 males, 5 females) sought help for all their symptoms, 10 (5 males, 5 females) sought no help, and 6 (4 males, 2 females) sought help for some symptoms. Table 1 provides an overview of the baseline characteristics (gender, age, body mass index, educational level, living situation, and for female participants, having children and type of delivery) and help-seeking status for each PFS.

**Table 1:** Participant characteristics and symptoms by help-seeking behaviour.

Partici-pant number	Gender	Age (years)	Body mass index	Educational level	Living situation	Children	Type of delivery	LUTS		Anorectal dysfunction		Sexual dysfunction		Pelvic pain		Pelvic organ prolapse	
								Help	No help	Help	No help	Help	No help	Help	No help	Help	No help
1	M	78	25.1	lower	living with partner			X		X							
2	M	70	30.7	medium	living alone				X			X					
3	M	82	23.8	lower	living with partner				X		X						
4	M	66	32.5	higher	living with partner				X			X					
5	M	86	25.2	lower	living with partner				X				X				
6	M	71	19.0	higher	living with partner				X		X						
7	M	41	30.3	medium	living with partner and children						X		X				
8	M	54	31.4	lower	living with partner				X				X				
9	M	64	27.2	higher	living with partner			X		X							
10	M	62	24.8	higher	living with partner			X					X				
11	M	67	29.5	lower	living alone			X		X		X					
12	M	74	26.4	lower	living alone			X		X				X			
13	M	78	26.0	medium	living with partner			X			X	X					
14	F	66	22.6	lower	living with partner	yes	2 VD	X		X			X	X		X	
15	F	77	30.1	lower	living with partner	yes	2 VD	X						X			
16	F	50	31.3	medium	living with partner and children	yes	2 VD, 1 CS		X		X						
17	F	67	26.8	medium	living with partner	yes	3 VD	X		X				X		X	
18	F	73	28.3	medium	living alone	yes	3 VD	X		X				X		X	
19	F	66	31.6	lower	living with partner	yes	2 VD	X						X		X	
20	F	68	27.4	lower	living with partner	yes	2 VD	X		X		X		X			
21	F	47	22.5	lower	partner, living with child	yes	1 VD		X						X		
22	F	63	18.8	lower	living with partner	no			X		X						
23	F	72	28.2	lower	living alone	no			X		X						
24	F	60	23.0	medium	living with partner	yes	3 VD				X		X				
25	F	52	26.8	higher	living with partner	no			X	X							

Abbreviations: CS, caesarean section; F, female; LUTS, Lower urinary tract symptoms; M, male; VD, vaginal delivery.

### 3.1 Factors associated with help-seeking behaviours

**Fig. 1:**
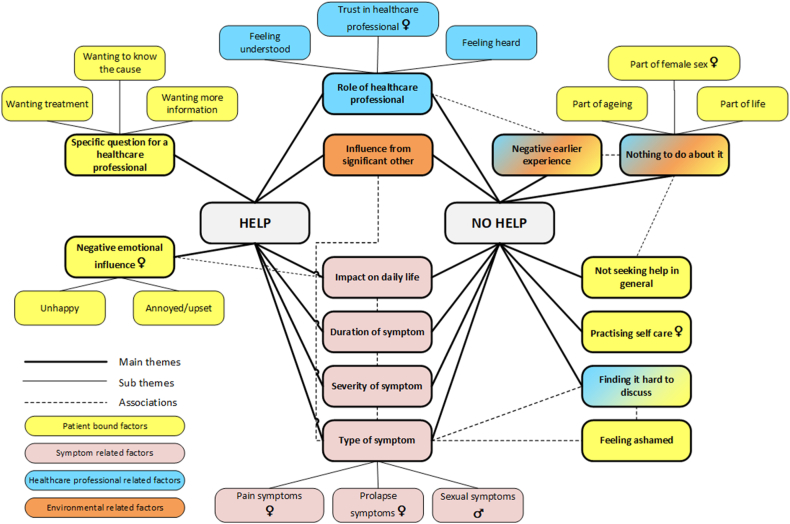
Facilitators and barriers associated with help-seeking behaviour among males and females with various pelvic floor symptoms. Facilitators are shown on the left and barriers are shown on the right. Themes that can be both facilitators and barriers are positioned in the middle. Factors related more to one of the sexes are noted with ♀ for females and ♂ for males. All other factors apply to both sexes.

The main factors associated with seeking professional help were categorized as being related to the patient, professional, environmental, or symptom. Fig. 1 shows the interactions between the themes and subthemes for each factor. The facilitators and barriers associated with seeking help are illustrated with quotes in Table 2 and Table 3, respectively.

**Table 2:** Facilitators of seeking help among males and females with at least two pelvic floor symptoms.

Factor	Theme	Subtheme	Quote
Patient	Having a specific question for their healthcare professional	− Wanting to know the cause of their symptoms − Wanting more information about their symptoms − Wanting treatment	*Participant 25 (F):* “Now was the time that I wanted to do something about my symptoms, and with that in mind I went to the GP and he just gave me the authorization.”
Patient	Negative emotional influence	− Annoyed/upset − Unhappy	*Participant 14 (F):* “But at a certain point I was so done with being wet every time. Yes, mostly the stool was troubling for me. You feel like a uh, a kid.”
Healthcare professional	Role of the healthcare professional	− Trusting their healthcare professional − Feeling understood − Feeling heard	*Participant 23 (F):* “I trust my doctor. I am not afraid of him. I have a good connection with him. I can say everything to him.”
Environment	Influence of significant others		*Participant 7 (M):* “She tells me: what you have is not normal, just go to the GP and get his opinion. It helps me that my partner stimulates in this.”
Symptom	Impact on daily life		*Participant 17 (F):* “I stopped playing tennis, I don't go running, and did not lift the children anymore. All that kind of things, everything where there is more pressure, you don't do anymore.“
Symptom	Duration		*Participant 4 (M):* “But at some point, it will take too long. It can happen one time or over time. But if that's going to take too long, then I'm talking in terms from many months to a year, two years.”
Symptom	Severity		*Participant 9 (M):* “The main reason to seek help was because I had very severe miction problems, I barely could pee anymore. It did hurt a lot.”
Symptom	Type of symptom		*Participant 17 (F):* “When the bladder came out, I did seek help because that scared me.”

Abbreviations: F, female; GP, general practitioner; M, male.

**Table 3:** Barriers to seeking help among males and females with at least two pelvic floor symptoms.

Factor	Theme	Subtheme	Quote
Healthcare professional/patient	Finding it hard to discuss the symptoms	− Feeling ashamed	*Participant 7 (M):* “… a little bit of shame … you are talking about your eh genitals and feces.”
Patient	Practicing self help		*Participant 10 (M):* “No, I did not seek help for that. I did uh … I did use tools and so on. So like a penis pump … to keep that going. Yes, in the long run, it worked.”
Healthcare professional/environment/patient	Having an earlier negative experience		*Participant 18 (F):* “I was so done with hospitals and having all tests done, that I was like let it be …. I lost my trust in different treatment options.”
Healthcare professional/environment/patient	Nothing to do about it Set ideas	− Part of life − Part of age − Part of (female) sex	*Participant 2 (M):* “Er yes, like I just said, I think that it is a symptom of the older male, there is little to do about it.” *Participant 17 (F):* “Well, yeah, it is a symptom of the female sex, why should you go to a doctor with it?”
Patient	Not seeking help in general		*Participant 3 (M):* Yes, well, I don't want to burden my doctor with everything that is going on. I will not bother him with every small thing. Most things will resolve on their own.
Healthcare professional	Role of the healthcare professional	− Not trusting their healthcare professional − Not feeling understood − Not feeling heard	*Participant 14 (F):* “Normally I would go to my GP, but I don't feel taken seriously so I don't go to the GP that easy anymore. I find it more difficult.”
Environment	Influence of significant others		*Participant 21 (F):* “I also heard negative stories from others saying: ‘Oh, it will not help’ so …”
Symptom	Duration		*Participant 19 (F):* “I think maybe it will pass by, or it will improve. You should not go back with the first pain you get, it might be over in a week or maybe two.“
Symptom	Severity		*Participant 16 (F):* “Mainly because I personally don't think it bothers me enough or eh frequent enough to seek help.”
Symptom	Impact on daily life		*Participant 1 (M):* “Yes, if there are, if there are really problems that hinder you, then I would start looking for help. But I don't have that.”
Symptom	Type of symptom		*Participant 14 (F):* “Also annoying to have to explain (sexual symptoms) and I know that I just immediately get emotional and I just find that more difficult. I find it difficult. I can go to the doctor with a pee. But I just find this difficult.”

Abbreviations: F, female; M, male.

#### 3.1.1 Symptom type and significant others

Participants expressed differences in the ease with which they could discuss different symptoms. For example, although most PFS are usually discussed with a partner, LUTS may also be discussed with friends or family, and defecation symptoms are rarely discussed at all.

#### 3.1.2 Symptom type and feelings of shame

When participants felt shame about their symptoms, they found it harder to discuss them with a medical professional or with others. Shame was strongly associated with defecation and sexual symptoms, which were also the most difficult to discuss.

“Yes, of course a little bit of the feeling of shame. Because you're talking about your uh genitals and uh about uh feces. And that is …, there is a little bit of a blockage, yes, quite a bit. Look, if I have a cut on my finger that won't heal properly then … I'd rather go to the doctor then with such a problem." (Participant 7, male).

#### 3.1.3 Negative experiences and the belief that nothing can be done

When participants had sought help in the past, they felt unheard if nothing was done about their symptoms or the physician did not discuss them. The sense that their symptoms could not be treated was confirmed if people around them also received ineffective treatment.

#### 3.1.4 Not seeking help in general and the belief that nothing can be done

Some participants indicated that they tend not to seek help routinely, and that not seeking help for their PFS simply reflected a continuation of this position. They mentioned not wanting to bother their healthcare professional and preferring to wait to see if symptoms resolved. We also encountered the fixed belief that PFS are a normal part of life that healthcare professionals cannot change.

“No, I only assumed that for the sexual complaints and the urinary complaints, well, it's an old man's complaint, so eh, there probably isn't much that can be done about that.” (Participant 2, male).

#### 3.1.5 Discussion difficulties and the healthcare professional's role

Several participants considered their PFS to be private and found them difficult to discuss. The sex of the healthcare professional did not affect their decision to seek help, but having a good relation with them was a clear facilitator. Knowing that the healthcare professional will listen and take their symptoms seriously facilitated them seeking help.

### 3.2 Comparison between males and females

Males and females agreed on most factors and themes, but not all. Before or during the help-seeking process, 11 females indicated that they had attempted self-care, including the use of incontinence materials, lubrication, or pelvic floor exercises (e.g., based on internet research) to reduce the impact of symptoms on their life. Although males indicated that they had practiced self-care, they did so to a lesser extent than females. Additionally, several females reported finding it difficult to talk about sexual symptoms, while participating males expressed greater ease talking about these symptoms with either a doctor or a partner.

“Also annoying to have to explain (sexual symptoms) and I know that I just immediately get emotional and I just find that more difficult. I find it difficult. I can go to the doctor with a pee. But I just find this difficult.” (Participant 14, female).

Females indicated more often that their PFS had negative emotional effects and were bothersome. This greater reported severity was a strong facilitator of seeking help. Another subtheme specific to females was the sense of futility when seeking help, with many holding the belief that PFS are a normal part of being female. Nevertheless, having POP or pain served as reasons for females seeking help, and it seemed particularly important for them to trust their healthcare professional. Those with a negative past encounter experienced this as a barrier to seeking further help.

By contrast, males indicated the presence of sexual symptoms as being very bothersome and sought help for them more often than females. Males also reported a greater dependence on significant others, such as partners and friends, for advice on when to seek help. Whereas females focused on the negative experiences of family and friends to avoid seeking help, males used the input of family and friends as a prompt to seek help.

“Yes, that helps too. Look, when she's pushing, well, um … She reflects for me and she says: yes, what you have now is not normal. Just go to the doctor, then you can hear what the GP thinks. Then you know whether it is something serious or … or not. Yes, it helps that a partner encourages me in this.” (Participant 7, male).

## 4 Discussion

This exploratory study of help-seeking behaviour by males and females with multiple PFS confirmed known barriers, such as embarrassment, thinking the condition is not serious enough, or perceiving PFS as a normal part of aging [12,17] and facilitators, including experiencing bothersome or worsening symptoms and knowing that treatment options exist [12,17]. However, our study showed that differences in help-seeking exist within one person depending on the type of symptom. Furthermore, our study revealed many similarities, but also some distinct differences, between the sexes. For example, consistent with other research, females engaged in self-care more often than males. This could reflect the fact that females are more familiar with pad use [27]. However, other differences showed varying levels of agreement with the existing literature.

Notably, males stated they did not really need to seek help for PFS and that they could wait until the symptoms had a bigger impact on their daily life. An increasing impact on quality of life certainly appears to be a strong facilitator in elderly males with LUTS [13]. Nevertheless, our finding that males do not experience their own symptoms as severe enough to warrant a consultation, preferring not to bother their doctor, contrasts with two earlier studies on gender differences that reported males with UI sought help for less serious symptoms than females [11,12]. This could be explained by the broader scope of our study and the inclusion of concurrent PFS. Although males and females both report sexual symptoms as bothersome, few appear to seek help [14]. In the present study, having sexual symptoms could serve as either a barrier to or a facilitator of seeking help, with a greater facilitating effect for males than for females.

Females stated the importance of trusting their healthcare professional, which served as both a barrier and facilitator in relation to seeking help for PFS. Females clearly do not seek help when they believe nothing can be done, they have had negative experiences, or they feel their complaint was not taken seriously.

Having pain or prolapse symptoms substantially affect daily life and can lead to females seeking help. This finding agrees with that of Morrill et al., who reported that females were most likely to seek care for POP than for UI and defecation problems [10]. In addition, patients in the current study more often reported that pain complaints resulted from earlier treatment for another pelvic problem (e.g., surgical mesh placement). These females had already been in contact with a healthcare professional and were actively asked about symptoms that occur after specific surgeries. Given that this group was already in the patient role, they did not need to justify a new consultation. Moreover, healthcare professionals in these settings had probably received specific training to monitor the side effects associated with those surgeries.

A recent study by our research group that focused on concomitant PFS found that many males and females complaining of pelvic pain have at least one other PFS [4]. The current interview-based study adds that the presence of pain led to most participants seeking help. The only participant who did not seek help for pain symptoms did not seek help for any PFS because she found them difficult to discuss.

This research benefited from the use of purposive sampling to inventory the diverse arguments for seeking and avoiding help. By inviting participants who sought help for some PFS, we gained important insights into both the intra- and inter-personal variation in these arguments. Nevertheless, there was still an underrepresentation of males with pain symptoms, whereas pain was a frequent symptom among females. Although this could have led to differences in reported help-seeking behaviour between males and females, it reflects the sex differences in response to pain that have been reported elsewhere [4,28]. Moreover, we reached saturation for both males and females, suggesting that we did not miss any relevant items.

Several weaknesses also warrant attention. Using a semi-structured interview could lead to undue influence by the interviewer on the course of the interview and the results.Furthermore, participants were not able to choose between a female or male interviewer as two female researchers conducted the interviews. Although conceivable that men find it more difficult to talk openly to a female interviewer, we could not find any evidence for that in literature. Moreover, based on our interview data we don't have the impression that our male participants suppressed certain topics. The interviews were also conducted by telephone, removing the ability to assess non-verbal communication; however, this will also have increased the perception of anonymity, which could have made it easier for participants to talk about their symptoms and behaviours [29]. Another point of attention is the age of our participants. Despite purposive sampling on age, the mean age of our sample was relatively high, with the mean age of males being slightly higher than that of female participants. A recent study on predictors of help-seeking behavior in women with urinary incontinence found that when age increased, help-seeking was decreasing [30]. Therefore, it could be that the older participants in our study were more reluctant to seek help, compared with younger participants. We found an indirect confirmation of this relation as the elderly people more often considered PFS as part of ageing and did not want to bother the GP with their complaints compared with younger participants. The type of parenting and upbringing and the general thoughts about PFS in earlier days might play a role in this.

As this is qualitative research, we can't say anything about the association in general between the participant characteristics (such as gender, age, educational level) and the mentioned facilitators and barriers. Finally, the risk of unintentional selection cannot be discounted in this type of research. The interviewees were unavoidably selected based on their willingness to discuss complaints with a stranger, which could introduce bias.

## 5 Conclusion

Males and females with multiple PFS share many help-seeking behaviours, though differences exist for some symptoms. Irrespective of the sex, healthcare providers should be aware that patients seeking help for one problem likely have a concurrent symptom for which they are not seeking help.

## Data availability statement

The data that support the findings of this study are available from the corresponding author, upon reasonable request.

## Funding statement

This study was funded by 10.13039/501100001826ZonMw (Gender and Health 849200004).

## Ethical statement

The study was approved by the local medical ethical committee (University Medical Center Groningen: METc2018/601). All participants provided written informed consent.

### Clinical trial registration

The study is registered at ClinicalTrials.gov, number NCT03558802.

## CRediT authorship contribution statement

**Kim Groot Wesseldijk:** Formal analysis, Validation, Visualization, Writing – original draft. **Hannah E. van Reemst:** Writing – review & editing, Investigation, Formal analysis, Data curation. **Ellen ter Horst:** Writing – review & editing, Investigation, Formal analysis, Data curation. **Grietje E. Knol-de Vries:** Methodology, Investigation, Formal analysis, Data curation, Conceptualization, Project administration, Supervision, Visualization, Writing – review & editing. **Marco H. Blanker:** Writing – review & editing, Visualization, Conceptualization, Funding acquisition, Methodology, Supervision.

## Declaration of competing interest

The authors declare that they have no known competing financial interests or personal relationships that could have appeared to influence the work reported in this paper.
